# Nonlinear Analysis of Motor Activity Shows Differences between Schizophrenia and Depression: A Study Using Fourier Analysis and Sample Entropy

**DOI:** 10.1371/journal.pone.0016291

**Published:** 2011-01-28

**Authors:** Erik R. Hauge, Jan Øystein Berle, Ketil J. Oedegaard, Fred Holsten, Ole Bernt Fasmer

**Affiliations:** 1 Olaviken Psychiatric Hospital, Bergen, Norway; 2 Division of Psychiatry, Haukeland University Hospital, Bergen, Norway; 3 Department of Clinical Medicine, Section for Psychiatry, Faculty of Medicine and Dentistry, University of Bergen, Bergen, Norway; The Research Center of Neurobiology-Neurophysiology of Marseille, France

## Abstract

The purpose of this study has been to describe motor activity data obtained by using wrist-worn actigraphs in patients with schizophrenia and major depression by the use of linear and non-linear methods of analysis. Different time frames were investigated, i.e., activity counts measured every minute for up to five hours and activity counts made hourly for up to two weeks. The results show that motor activity was lower in the schizophrenic patients and in patients with major depression, compared to controls. Using one minute intervals the depressed patients had a higher standard deviation (SD) compared to both the schizophrenic patients and the controls. The ratio between the root mean square successive differences (RMSSD) and SD was higher in the schizophrenic patients compared to controls. The Fourier analysis of the activity counts measured every minute showed that the relation between variance in the low and the high frequency range was lower in the schizophrenic patients compared to the controls. The sample entropy was higher in the schizophrenic patients compared to controls in the time series from the activity counts made every minute. The main conclusions of the study are that schizophrenic and depressive patients have distinctly different profiles of motor activity and that the results differ according to period length analysed.

## Introduction

Assessing the motor activity of the patients has always been an integral part of a psychiatric evaluation. Yet the possibility of an objective registration of the motor activity as it varies over hours and days seems not to have lead to its widespread use in clinical practice, neither in diagnosis nor for monitoring symptom severity or treatment effect. This is in contrast to other fields of medicine, for instance neurology, where objective motor registration is employed in the investigation of sleep patterns and diurnal rhythm of symptoms [1] [2], as well as in the investigation of movement disorders [3] and the dementias.

During the last decade, a new framework for analysis and evaluation of time series has emerged. Methods from nonlinear dynamics have been applied to a number of subjects encompassing numerous areas in physiology and clinical medicine [4] [5] [6] [7] [8] [9] [10]. These methods range from studying the randomness versus chaos by measuring the nonlinearity in a signal to estimating the fractal dimension of its corresponding attractor. A number of researchers have, by using different algorithms, studied the entropy of a time series i.e. different measures of the order and predictability in the series [11]. Basic information of nonlinear analysis as well as various purposes of applications can be found in the references mentioned above.

In neuropsychiatry, these nonlinear methods have been applied to mood variations [12], EEG-recordings in a number of conditions [13] and to test neurobehavioral and neuropsychologic measurements in schizophrenic patients [14].

In this study we wanted to investigate the patterns of motor activity in two important psychiatric diagnostic groups, patients with schizophrenia and patients with depression, and to compare these two groups to each other and to a control group of healthy volunteers. These patients have previously been studied by us [22]. Then, we recorded average motor activity and three variables: interdaily stability (IS), intradaily variability (IV), and relative amplitude (RA). One of the findings was that the schizophrenic patients had higher IS and lower IV than the controls in contrast to the depressive patients. The current study investigates the motor activity further with quite different methods. The aim was twofold. First we wanted to describe the motor activity data better, using classical methods, and second we wanted to use Fourier analysis and entropy measurements to see if and how the study groups differed from each other. The main hypothesis to be tested was to see if technical recording of motor activity could provide objective, valid and valuable data in the assessment of these frequently observed symptoms in patients with psychiatric disorders.

## Methods

### Ethics statement

The study protocol was approved by the Norwegian Regional Medical Research Ethics Committee West. Written informed consent was obtained from all participants involved in the study.

### Subjects

The study group consisted of 24 chronic psychotic patients (3 women and 21 men), all with a diagnosis of schizophrenia, from an open ward for long-term patients (Knappentunet), 25 patients with mood disorders (11 women and 14 men), all currently depressed, five inpatients (20%) from an open psychiatric ward and 20 outpatients (80%), all from the University Hospital of Bergen. Control subjects were medical students (n = 5), patients without serious medical or psychiatric symptoms from a primary care office (n = 4) and employees from Knappentunet (n = 23). The control group consisted of 20 women and 12 men, average age 38.2±13.0 years (mean ± SD), range 21–66. None of the control subjects had a history of affective or psychotic symptoms.

The 25 patients with major depression had mean age 42.9±10.7 years and their mean education was 11.6±2.8 years. Their mean MADRS score at the start and end of registration were 22.8±4.6 and 19.6±5.4, respectively. Of the patients, 14 (56%) were male, 11 (44%) were married/cohabitating and 4 (16%) were employed. Their diagnoses are shown in Table 1. In Table 2 the current drug treatments of the patients with major depression are listed. In the group of 6 patients who received antipsychotics are included 2 patients who were treated with small doses of antipsychotics at bedtime.

**Table 1:** Diagnoses of patients with a major depressive episode (n = 25).

Diagnosis	N (%)
Unipolar major depr.disorder	18 (72%)
Bipolar I disorder	1 (4%)
Bipolar II disorder	7 (28%)

**Table 2:** Psychotropic drug treatment for patients with major depression (n = 25).

Treatment	N (%)
Antidepressants	17 (68%)
Lithium	5 (20%)
Mood stabilizers except lithium	1 (4%)
Antipsychotics	6 (24%)
Anxiolytics	3 (12%)
Hypnotics	2 (8%)
No psychotropic drug treatment	8 (32%)

The group of 24 patients with schizophrenia had mean age 47.4±11.1 years (range 27–69 years). Their mean age at first hospitalization was 24.4±9.3 years (range 10–52 years). Of the patients, 21 (88%) were male and 18 (75%) had a paranoid form of schizophrenia. We were able to obtain BPRS scores in 19 of the 24 schizophrenic patients, with a mean of 51.5±9.5 (range 34–68). The current drug treatments of these patients are shown in Table 3. For all the patients who were treated with clozapine therapeutic drug monitoring (TDM) were performed. Their mean clozapine dose was 481±218 mg (range 300–900 mg), and a mean serum level of clozapine 1671±1164 mmol/l (range 326–3333 mmol/l) were measured.

**Table 3:** Psychotropic drug treatment for patients with schizophrenia (n = 24).

Treatment	N (%)
Traditional antipsychotics	6 (25%)
Atypical antipsychotics except clozapine	9 (37%)
Clozapine	9 (37%)
Mood stabilizers	5 (21%)

The sum exceeds n (100%) as some patients receive more than one type of treatment.

### Diagnostic procedures

For the diagnosis of patients with depressive disorders we used a semi-structured interview based on DSM-IV criteria (American Psychiatric Association 1994) for mood disorders [15]. All diagnostic assessments were performed by one of the authors (OBF). Diagnostic evaluations of the chronic psychotic patients were made by one of the authors (JØB) and a consensus diagnosis, based on DSM-IV criteria, was made after discussion of each case with OBF. The depressive and psychotic symptoms were evaluated by the use of the Montgomery-Asberg Depression Rating Scale (MADRS, [16] and the Brief Psychiatric Rating Scale (BPRS, [17], respectively.

### Recording of motor activity

Motor activity was monitored with an actigraph worn at the right wrist (Actiwatch, Cambridge Neurotechnology Ltd, England). In the actigraph, activity is measured by using a piezo-electric accelerometer that is programmed to record the integration of intensity, amount and duration of movement in all directions. A corresponding voltage is produced and is stored as an activity count in the memory unit of the actigraph.

The right wrist was chosen to make the procedure more convenient for the participants, since most of them have their watches around the left wrist and it is cumbersome to have two such devices on the same arm. Previous studies have shown that there are small differences between the right and left wrist [18], [19]. Total activity counts were recorded for one minute intervals for a continuous period of two weeks. Both patients and controls were instructed to wear their actigraphs at all times except when taking a shower, and to note the periods when the actigraph was taken off the wrist.

Because many of the schizophrenic and depressive patients had shorter and more prolonged periods of inactivity, we searched manually each time series to find periods with continuous motor activity. From each participant we selected a period with a 300 min, containing not more than 4 consecutive minutes with zero activity, by searching from the start of the series and using the first period that satisfied this criterion. In this way we were able to obtain 300 min sequences from each participant. In addition data were analyzed using information from the whole two week period, with activity count for one hour as the unit of measurement.

### Mathematical analyses

The software used in the different analyses were found at the PhysioToolkit Research Resource for Complex Physiologic signals; see http://www.physionet.org [20].

#### Sample entropy

Data were normalized before analysis, by transforming the time series to have sample mean 0 and sample variance 1. Sample entropy is a nonlinear measure, indicates the degree of regularity (complexity) of time series, and is the negative natural logarithm of an estimate of the conditional probability that subseries of a certain length (m) that match point-wise, within a tolerance (r), also match at the next point. We chose m = 2 and r = 0.2. Sample entropy was employed since it is regarded as the best measure of entropy in biological time series [21].

#### Fourier analysis

Data were normalized before analysis. No windows were applied. For analysis of the data from the 300 min periods, the first 256 min were used. Results are presented as variance (% of the total variance) in four components of the spectrum: 0,0000651–0,00104167 Hz (corresponding to the period from 16–256 min); 0,00110677–0,00208333 Hz (8–15 min), 0,00214844–0,00416667 Hz (4–8 min), and 0,00423177–0,00833333 Hz (2–4 min). For analysis of data for the whole 2 weeks period, activity counts for one hour periods were used, from the first 256 hours of the recordings. Results are presented as the relation between variance in the low frequency part of the spectrum (corresponding to the periods 26–256 hours) and the high frequency part (corresponding to 2–21 hours), omitting the variance attributed to the 24 hour peak.

### Statistics

One-way ANOVA was used. We used the Bonferroni correction when performing the subsequent pairwise comparisons of the differences between groups. Pearson's correlation coefficient was employed when evaluating correlations. SPSS version 15.0 was used for the statistical analyses.

## Results

**Figure 1:**
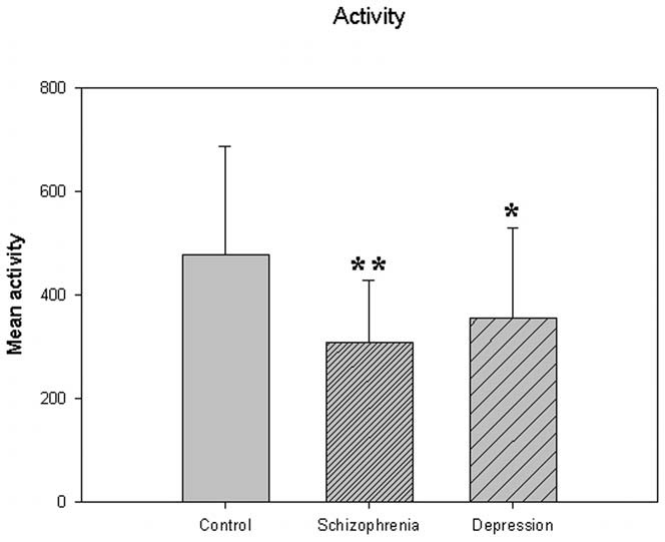
Mean activity from **Table 4**. *p<0.05, **p<0.01 compared to control group.

**Figure 2:**
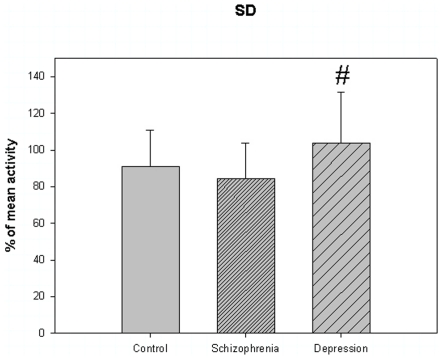
Standard deviation from **Table 4**. # p<0.05 depression compared to schizophrenia.

**Figure 3:**
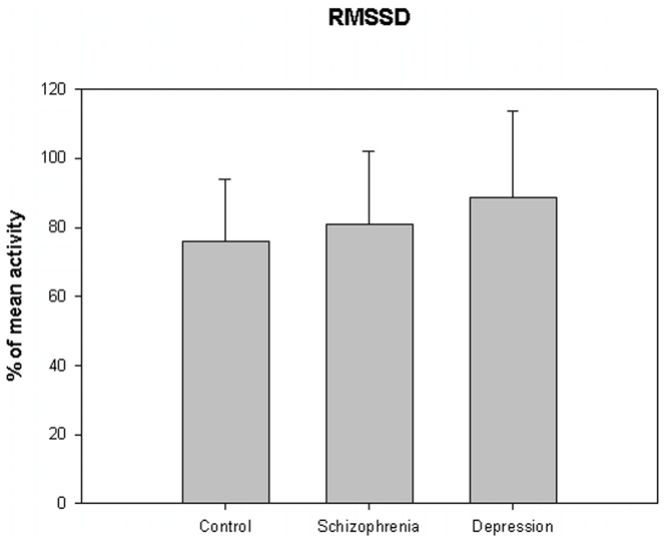
Root mean square successive differences from **Table 4**.

**Figure 4:**
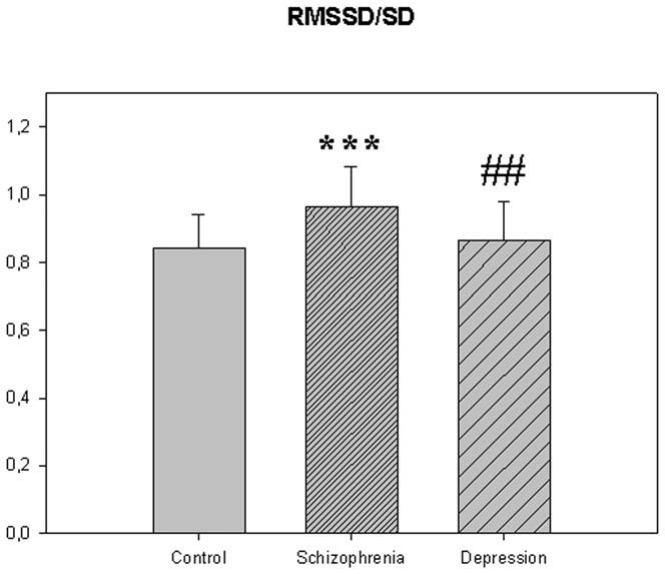
RMSSD/SD from **Table 4**. *** p<0.001; schizophrenia compared to the control group. ## p<0.01; depression compared to schizophrenia.

**Figure 5:**
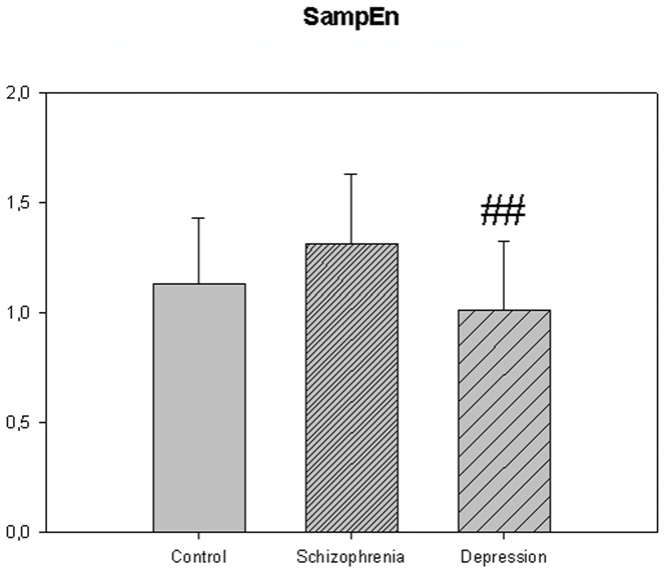
Sample entropy from **Table 4**. ## p<0.01; depression compared to schizophrenia.

**Figure 6:**
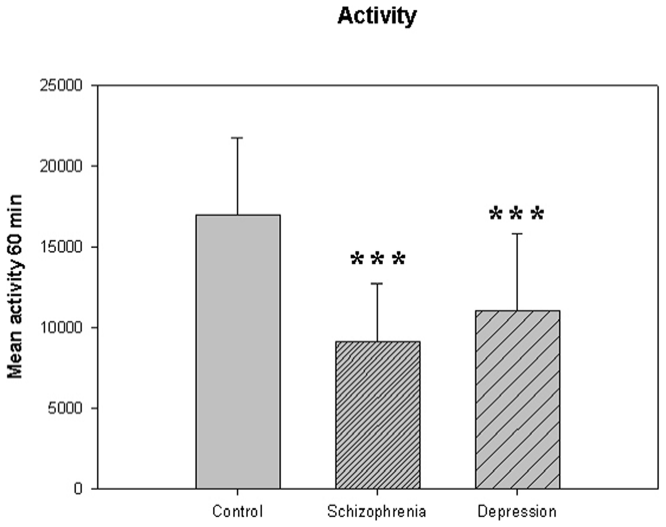
Activity from **Table 5**. *** p<0.001; schizophrenia and depression compared to the control group.

**Figure 7:**
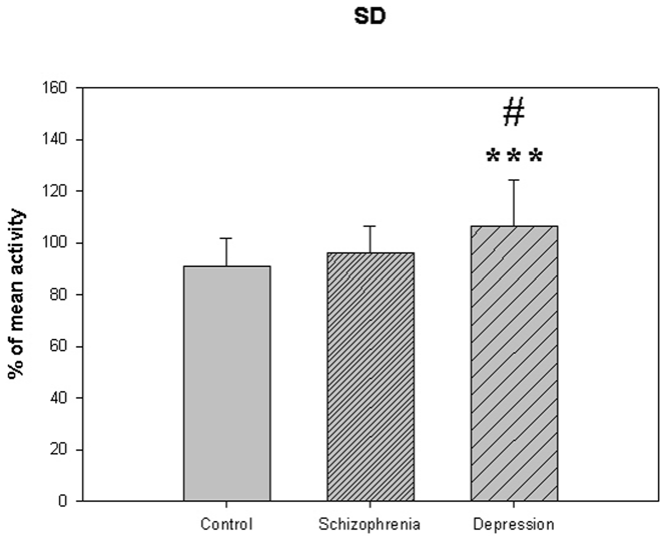
Standard deviation from **Table 5**. *** p<0.001; depression compared to the control group. # p<0.05 depression compared to schizophrenia.

**Figure 8:**
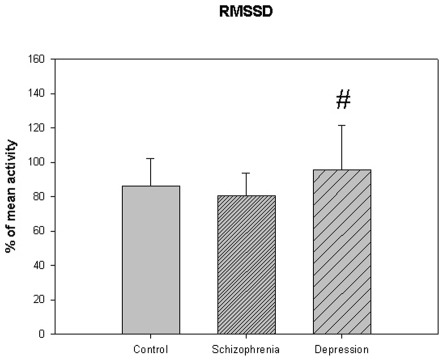
Root mean square successive differences from **Table 5**. # p<0.05 depression compared to schizophrenia.

**Figure 9:**
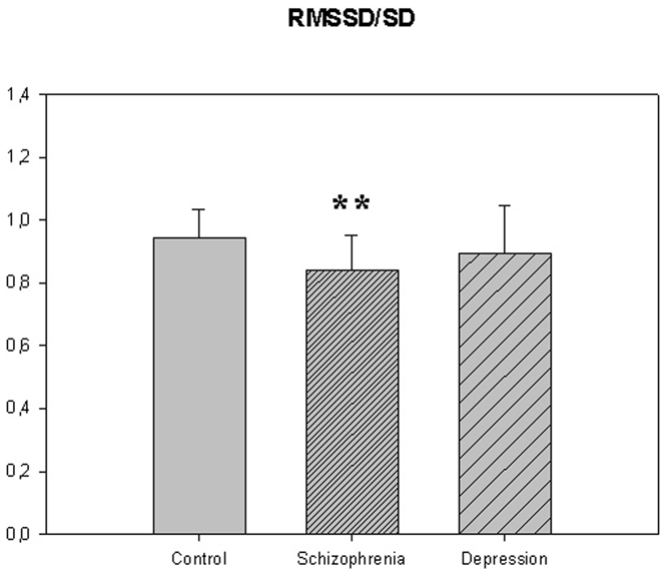
RMSSD/SD from **Table 5**. **p<0.01 schizophrenia compared to control group.

**Figure 10:**
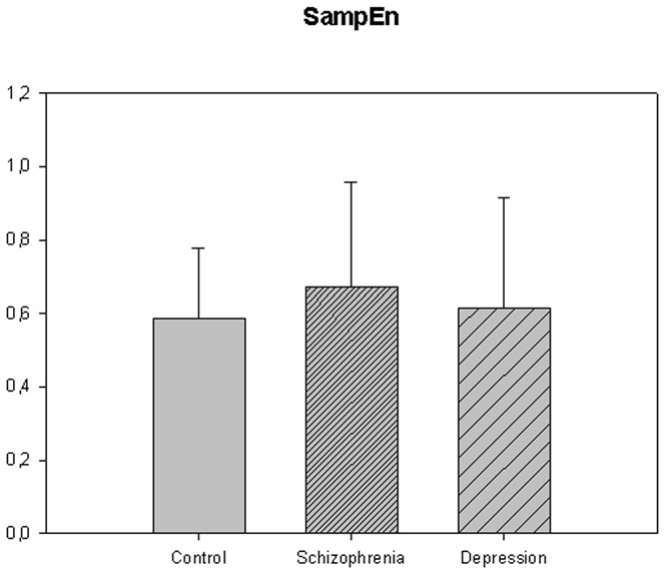
Sample entropy from **Table 5**.

**Figure 11:**
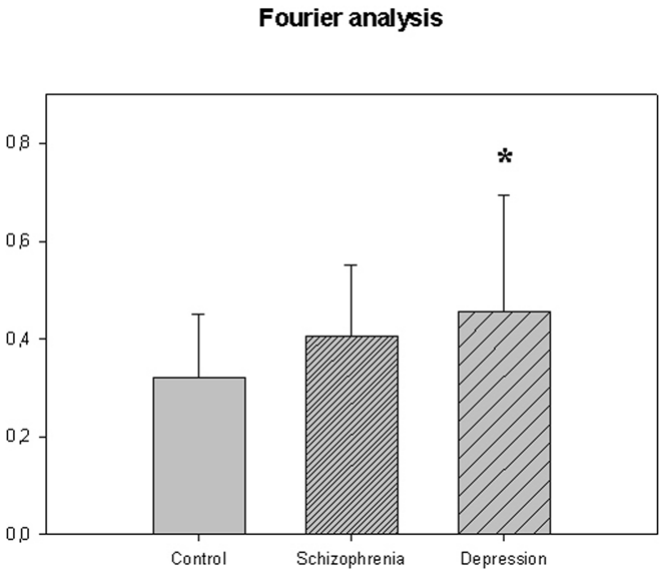
Fourier analysis from **Table 5**. *p<0.05, depression compared to control group.

The motor activity was significantly lower in the schizophrenic patients and in patients with major depression, compared to controls (Table 4, Figures 1, 2, 3, 4, 5 for 300 min periods, and Table 5, Figures 6, 7, 8, 9, 10, 11 for 2 weeks periods). The differences between schizophrenic and depressive patients were not significant. The standard deviations (SD) in the three groups were significantly different (ANOVA) both for the 300 min periods (Table 4) and the 2 weeks periods (Table 5). In both cases the depressive patients had the highest SD, significantly different from the schizophrenic patients for the 300 min periods and significantly different from both schizophrenic patients and controls for the 2 weeks periods. The root mean square successive differences (RMSSD) were also highest in the depressive patients, but significantly different from the schizophrenic patients only for the 2 weeks periods. However, when RMSSD was compared to the SD, this ratio (RMSSD/SD) was significantly higher in the schizophrenic patients than in both controls and patients with depression, when analyzing data from the 300 min periods (Table 4). In contrast, for the 2 weeks periods RMSSD/SD was lower in the schizophrenic patients compared to both controls and patients with depression (Table 5), but the difference was significant only for the comparison with controls.

**Table 4:** Results from actigraphic recording of 300 min periods with continuous motor activity.

	Control	Schizophrenia	Depression	ANOVA
Mean activity	479±208	309±119 **	355±174 *	F(78,2) = 7.196, P = 0.001
SD (% of mean activity)	90.9±19.8	84.2±19.7	103.7±27.6 #	F(78,2) = 4.836, P = 0.01
RMSSD (% of mean activity)	76.1±17.9	80.8±21.2	88.7±25.0	F(78,2) = 2.498, P = 0.089
RMSSD/SD	0.841±0.100	0.963±0.121 ***	0.863±0.115 ##	F(78,2) = 8.939, P<0.001
SampEn (m = 2, r = 0.2)	1.129±0.299	1.311±0.318	1.012±0.311 ##	F(78,2) = 5.858, P = 0.004

All data are given ± SD. Post hoc Bonferroni tests:

*p<0.05,

**p<0.01,

***p<0.001; schizophrenia or depression compared to the control group.

#p<0.05,

##p<0.01; depression compared to schizophrenia.

**Table 5:** Results from actigraphic recordings for 2 weeks.

	Control	Schizophrenia	Depression	ANOVA
**Mean activity (60 min)**	17031±4766	9097±3604 ***	11057±4732 ***	F(75,2) = 24.207, P<0.001
**SD (% of mean activity)**	90.8±10.9	95.9±10.5	106.2±18.2 ***#	F(75,2) = 8.931, P<0.001
**RMSSD (% of mean activity)**	86.1±15.9	80.6±13.1	95.5±25.9 #	F(75,2) = 3.760, P<0.028
**RMSSD/SD**	0.942±0.092	0.842±0.108 **	0.896±0.150	F(75,2) = 4.986, P<0.009
**SmpEn (m = 2, r = 0.2)**	0.588±0.189	0.670±0.287	0.616±0.297	F(75,2) = 0.702, P = 0.499
**Fourier analysis**	0.320±0.131	0.406±0.146	0.455±0.240 *	F(75,2) = 4.242, P = 0.018

All data are given ± SD. Results from the Fourier analysis are the ratios between variance in the low frequency (corresponding to periods 26–256 hours) and the high frequency (2–21 hours) parts of the spectrum.

Post hoc Bonferroni tests:

*p<0.05,

**p<0.01,

***p<0.001; schizophrenia or depression compared to the control group.

#p<0.05; depression compared to schizophrenia.

**Figure 12:**
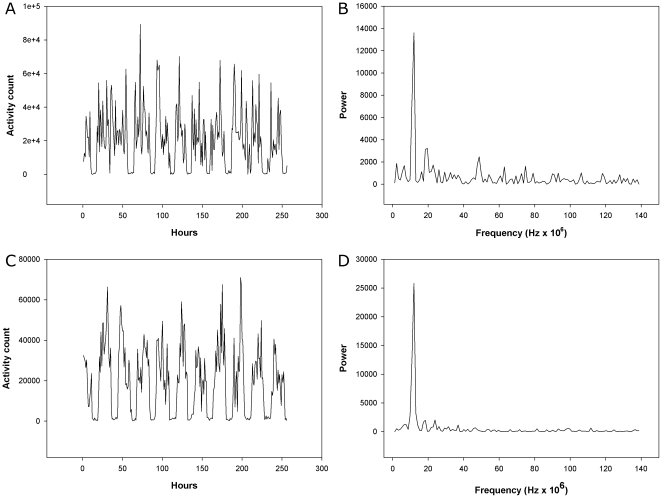
Actigraph recordings of 256 min periods. Activity counts and Fourier analysis from a control person (A and B) and a patient with schizophrenia (C and D).

Detailed findings from the Fourier analysis are given in Table 6. In Figure 12 are shown activity counts from 300 min periods from one control person and one patient with schizophrenia, and results from Fourier analyses. The Fourier analysis of the 256 min periods showed that the schizophrenic patients had a distinctly different pattern of variance compared to both controls and patients with depression. Variance in the two high frequency components of the spectrum (corresponding to 2–4 min and 4–8 min periods) were significantly higher in the schizophrenic patients compared to controls (29% and 40% higher) and patients with depression. In the low frequency part of the spectrum the schizophrenic patients had a significantly lower variance than controls (22% lower) and patients with depression. The depressed patients were not significantly different from controls.

**Table 6:** Fourier analysis of the actigraphic recording of 300 min periods with continuous motor activity.

Period	Control	Schizophrenia	Depression	ANOVA
2–4 min	15.4±5.0	19.8±5.8 *	15.9±6.1#	F(78,2) = 4.773, P = 0.011
4–8 min	13.8±3.9	19.3±4.0***	15.4±3.9 ##	F(78,2) = 14.154, P<0.001
8–15 min	14.7±4.7	17.1±4.5	16.0±5.2	F(78,2) = 1.827, P = 0.168
16–256 min	56.2±9.8	43.7±8.8 ***	52.8±10.8 ##	F(78,2) = 11.331, P<0.001

All data are given as % of total variance ± SD. Post hoc Bonferroni tests:

* p<0.05,

** p<0.01,

*** p<0.001; schizophrenia or depression compared to the control group.

#p<0.05,

##p<0.01; depression compared to schizophrenia.

**Figure 13:**
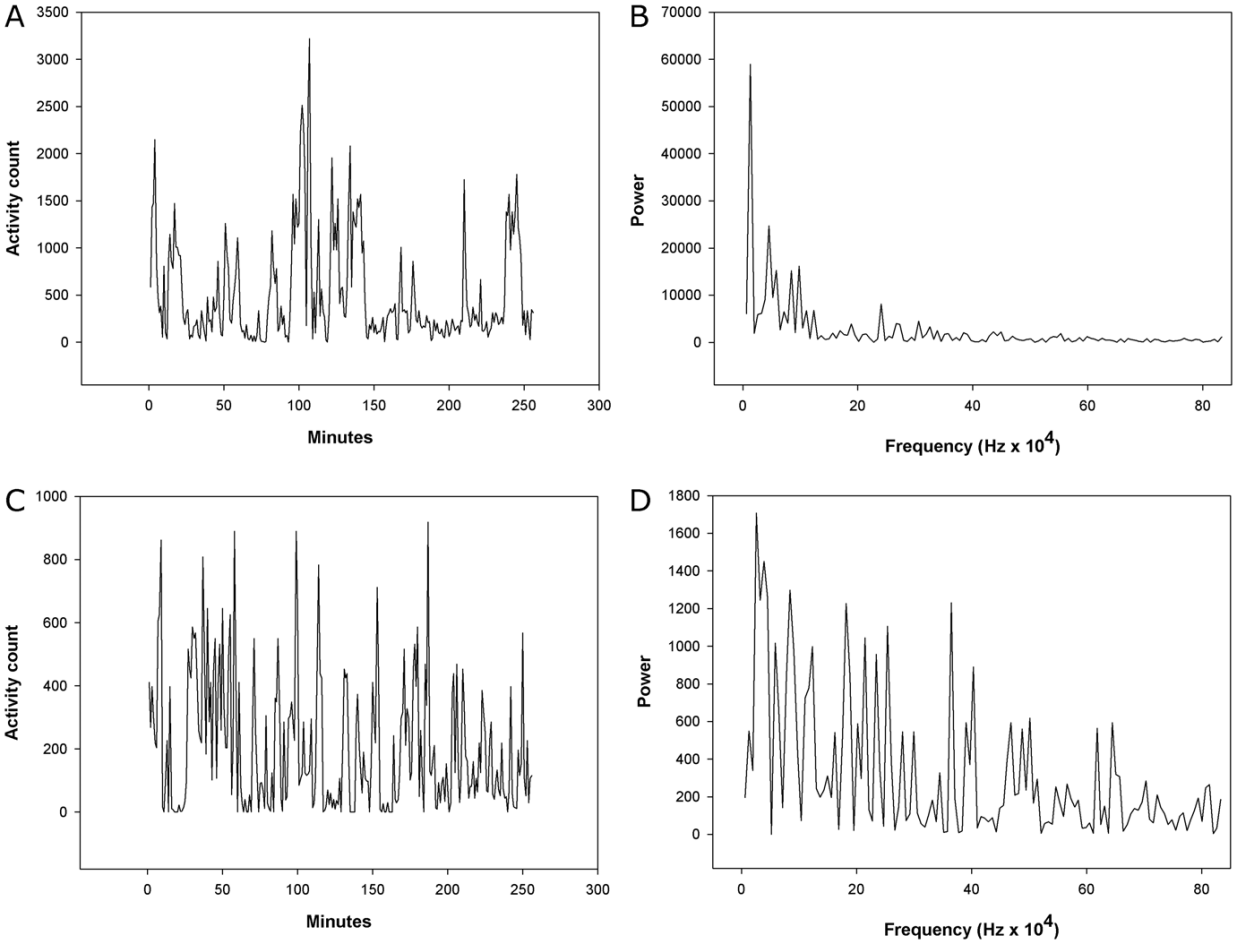
Actigraph recordings of 256 h periods. Activity counts and Fourier analysis from a control person (A and B) and a patient with depression (C and D).

In Figure 13 are shown activity counts from 256 hour periods from one control person and one patient with depression, and results from the Fourier analyses. Regarding the 256 hour periods, the Fourier analysis showed that the schizophrenic patients were not different from controls, while the depressed patients, when compared to controls, had a significantly higher ratio (42%) of variance in the low frequency (corresponding to the period 26–256 hours) and the high frequency part of the spectrum (2–21 hours).

The sample entropy was significantly higher in the schizophrenic patients compared to both controls (16% higher) and depressed patients (30% higher) for the 300 min periods. The sample entropy of the depressed patients was lower than for the controls, but not significantly so. For the 2 weeks periods the sample entropy results were not different across the three groups.

In Table 7 are shown correlations between sample entropy and the measures of variance we have used (SD, RMSSD, RMSSD/SD and Fourier analysis), analyzed separately in the three groups. For SD there are strong negative correlations in all the groups and for both 300 min and 256 hour series. Similarly, RMSSD values show a strong correlation to sample entropy, but only for the 300 min periods. The RMSSD/SD ratio for 300 min periods is correlated to sample entropy in the control group and in the depressed patients, but not in the schizophrenic patients. Fourier analyses in the schizophrenia group for 256 min periods and in the depression group for 256 hour periods show no significant correlations to sample entropy. (p between 0.194 and 0.987, t-test). When comparing the groups we therefore combined results from male and female participants. Average age in the schizophrenia group was significantly higher than in the control group, but when analyzed separately in these two groups there were no significant correlations with age, except for sample entropy analyses of 256 hour series for controls (0.391, p = 0.027).

**Table 7:** Correlations between sample entropy (2, 0.2) and various measures of variance.

		300 min	P	256 h	P
Control	SD	−0.760	<0.001	−0.479	0.006
	RMSSD	−0.513	0.003	−0.250	0.167
	RMSSD/SD	0.361	0.042	0.176	0.334
	Fourier analysis 4–8 min	−0.041	0.823		
	Fourier analysis 26–256 h/2–21 h			−0.199	0.275
Schizophrenia	SD	−0.756	<0.001	−0.567	0.005
	RMSSD	−0.632	0.001	0.093	0. 674
	RMSSD/SD	0.205	0.336	0.620	0.002
	Fourier analysis 4–8 min	0.246	0.247		
	Fourier analysis 26–256 h/2–21 h			−0.408	0.053
Depression	SD	−0.678	<0.001	−0.512	0.013
	RMSSD	−0.415	0.039	−0.271	0.211
	RMSSD/SD	0.463	0.020	0.157	0.473
	Fourier analysis 4–8 min	0.465	0.019		
	Fourier analysis 26–256 h/2–21 h			-0.321	0.135

The average time the actigraphs were taken off the wrist was not different for the three groups (1.8% of the total time in the control group, 1.3% in the depressed patients, and 0.6% in the schizophrenic patients).

## Discussion

The main finding of the present study is that schizophrenic and depressive patients have distinctly different profiles of motor activity when using linear and non-linear methods of analysis. These profiles are also different from healthy controls. As described previously [22] there was also a significantly reduced motor activity in both patient groups compared to the controls. This is in accordance with the clinical impression that people with chronic schizophrenia tend to live a more passive life and the fact that low activity is one of the most common signs of depression. Our study indicates, however, that this reduced level of activity is quite heterogenic. As can be easily seen, even using classical methods of analysis, subtle, but significant, differences can be found between the patient groups. The SDs in the registrations from patients in the depressive group were higher than the registrations from the schizophrenic patients and the controls, indicating more fluctuations in the activity level. Such increased intraindivual variability has been described in patients with ADHD [23], mostly in different neuropsychological tests [24], but also in actigraph recordings [25]. Furthermore, increased variability has been described in neuropsychological tests in patients with, both schizophrenia and depression [26]. Russel and collaborators [24] have put forward the hypothesis that increased variability in ADHD-patients may be related to impaired energy metabolism in the brain, and they suggest that this may also be relevant for other brain disorders.

Our results should be interpreted with caution. The representativeness of the patient groups can be questioned. The diagnoses of the patients in the study were all assessed non-blind, but on the other hand, the methods of investigating the motor activity did not require a subjective evaluation. The schizophrenic patients in this study were hospitalized and clearly have a more severe course of the disease compared to schizophrenic patients who with appropriate medication live a more normal and independent life. The depressive patients were, on the contrary, mostly out-patients. Obviously, also the patients used different medication. All the patients in the schizophrenic group used antipsychotics, while this was clearly not the case for the patients in the depression group.

When using methods of analysis that utilize the temporal aspects of the activity recordings, i.e. analysing the recordings as time series, other differences between the groups emerge. The significantly increased RMSSD in the depression group compared to the control group is consistent with the increased SD in this group.

One would naturally expect the RMSSD to be related to the SD for the time series in that a uniformly larger SD also would lead to a larger RMSSD. Noteworthy is therefore the highly significant increase in the RMSSD/SD ratio, from the 300 min recordings, in the schizophrenic group, meaning that the alteration between successive registrations increases. Similarly, results form the Fourier analysis of the same time series showed an increased variance in the high frequency part of the spectrum, corresponding to the periods of 2–4 and 4–8 min. It therefore seems that variance is increased in the frequency range of 0.0021–0.0167 Hz (1–8 min). To the best of our knowledge such a finding has not previously been reported, and we propose that this reflects a fundamental difference between the schizophrenic patients and the other groups.

However, when using a time scale of hours, the results were different. The RMSSD/SD ratio was reduced in the schizophrenic patients. In the Fourier analysis the only significant finding was an increased variability in the low frequency range in the depressed patients. This underscores the importance of using different time scales for the analyses.

Dissimilarities between the schizophrenic patients and the other groups were also evident in the results from the entropy measurements from the 300 min time periods. An increase in entropy indicates in itself a higher level of disorder and unpredictability in a time series. In the present study the highly significant increase in sample entropy for the group of schizophrenic patients compared both to the depressive patients and the controls may represent or mirror a partial breakdown in the structured normal activities of everyday life for the schizophrenic patients. This was neither seen in the control group nor in the depressive group, although the latter also had a reduction in general motor activity similar to that of the schizophrenic group. However, we did not find a similar increase in entropy of the 2 weeks recordings, using one hour as the unit of measurement.

Sample entropy showed a strong negative correlation to the SD. However, the sample entropy values were not correlated to the significant findings in the schizophrenic patients (the increased RMSSD/SD ratio and the increased variability in the high frequency range).

Despite the limitations which we have mentioned above, the key finding of our study is still valid: the apparently similar reduction in activity levels for the schizophrenic and depressive patients conceals in fact quite different properties that emerge when linear and nonlinear analyzing methods are applied.

As far as we know, studies using nonlinear methods on activity recordings have not hitherto been published. Thus we have no previous results we can relate the present findings directly to. There have, however, been a number of studies that have focused on the possibility of chaotic processes in patients with schizophrenia and depression.

One research group [27] studied mood ratings in depression and found a possible underlying chaotic process. A different group [28] studied mood ratings in affective instability and found that the patients showed more variability than controls on the Mean Squared Successive Difference, while the value of the Fractal Dimension was reduced. Thus the presence of affective instability was associated with reduced complexity. Mood recordings of depressed patients have been studied by Yeragani and collaborators [29]. A lower approximate entropy in a group treated with pemoline compared to fluoxetine and placebo was found. Moreover, in mood recordings, an increase in entropy in the month preceding a manic or depressive episode in bipolar patients have been found [30]. Our study of motor activity does not indicate that the depressed patients are different from controls in terms of complexity.

A number of studies have addressed by nonlinear methods the severe disturbances in neurocognitive functioning that is regarded as one of the core features of schizophrenia [31] [32] [33]. A correlation between activity and changes in brain structures using wrist worn actigraphy and magnetic resonance imaging have been found [34]. Some researchers [35] have proposed that an altered sequential or temporal architecture is a key feature of this disorder. Our findings seem to support this hypothesis.

We believe that the current study indicates that further investigations of time series of motor activity, with linear and nonlinear methods, will be important in the future, both for theoretical psychopathological considerations and for the diagnosis and treatment of psychiatric disorders. In particular, it could be possible to evaluate the motor activity of depressive and schizophrenic patients, thereby obtaining a measure that could rate the severity, monitor recovery and prevent relapse. Further research will obviously be necessary before practical applicability is established.
